# Effects of diabetes on myocardial infarct size and cardioprotection by preconditioning and postconditioning

**DOI:** 10.1186/1475-2840-11-67

**Published:** 2012-06-13

**Authors:** Takayuki Miki, Takahito Itoh, Daisuke Sunaga, Tetsuji Miura

**Affiliations:** 1 Second Department of Internal Medicine, Sapporo Medical University School of Medicine, South-1 West-16, Chuo-ku, Sapporo 060-8543, Japan

**Keywords:** Diabetes mellitus, Infarct size, Preconditioning, Postconditioning

## Abstract

In spite of the current optimal therapy, the mortality of patients with ischemic heart disease (IHD) remains high, particularly in cases with diabetes mellitus (DM) as a co-morbidity. Myocardial infarct size is a major determinant of prognosis in IHD patients, and development of a novel strategy to limit infarction is of great clinical importance. Ischemic preconditioning (PC), postconditioning (PostC) and their mimetic agents have been shown to reduce infarct size in experiments using healthy animals. However, a variety of pharmacological agents have failed to demonstrate infarct size limitation in clinical trials. One of the possible reasons for the discrepancy between the results of animal experiments and clinical trials is that co-morbidities, including DM, modified myocardial responses to ischemia/reperfusion and to cardioprotective agents. Here we summarize observations of the effects of DM on myocardial infarct size and ischemic PC and PostC and discuss perspectives for protection of DM hearts.

## Introduction

Cardiovascular diseases are the leading cause of death, accounting for approximately 30% of all deaths worldwide [1]. Among the various cardiovascular diseases, acute myocardial infarction (AMI) has a high rate of mortality, and infarct size is a primary determinant of prognosis in these patients. The only established and clinically approved method to limit infarct size is restoration of coronary blood flow by percutaneous coronary interventions (PCI), thrombolytic agents or coronary bypass surgery. However, our recent review of clinical infarct size data indicated that infarct size after current reperfusion therapy was larger than 75% of the area at risk and larger than 20% of the left ventricle in one fourth of AMI patients [2]. Lack of substantial myocardial salvage in those patients cannot be simply attributable to long symptom-to-reperfusion time, and involvement of reperfusion injury [3] is also possible. Nevertheless, significant percentages of AMI patients suffer from insufficient cardiac function and have a poor prognosis even after successful restoration of coronary flow [2]. Therefore, the establishment of a novel strategy to limit the extent of infarction during ischemia/reperfusion is of great clinical importance.

As a cardioprotective strategy, ischemic preconditioning (PC) has received much attention for its potent infarct size-limiting effect since its first report by Murry et al. in 1986 [4]. Zhao et al. found that “conditioning” with repetitive ischemia/reperfusion at the time of reperfusion also affords protection and named the phenomenon ischemic postconditioning (PostC) [5]. Laboratory investigations to date have clarified outlines of the intracellular mechanisms of PC and PostC [6-9]. In addition, several agents targeting signaling relevant to PC and PostC have been shown to act as PostC mimetics [6-8]. However, clinical benefits of PC, PostC and their mimetics were not always demonstrated in clinical trials [8,10]. The discrepancy between results of animal studies and clinical trials may be explained by notable differences between animal models of AMI and AMI in humans [2,10]. First, there are species differences in regulatory mechanisms of cardiomyocytes such as contribution of sarcolemmal Ca^2+^ ATPase to overall Ca^2+^ handling. Second, the timing of administration of the test agent was not exactly the same in the positive animal studies and negative clinical trials. Third, most of the patients with AMI were pretreated with pharmacological agents for their co-morbidities at the onset of AMI, whereas animals were untreated, except for anesthetic agents, before AMI in most of the previous studies. Pharmacological agents used for treatment of hypertension, dyslipidemia and/or diabetes mellitus (DM) potentially modify intracellular signaling relevant to cytoprotection and thus myocardial responses to PC, Post and PC mimetics. Fourth, AMI patients recruited to clinical studies were generally old and had concomitant diseases, whereas relatively young and healthy animals were used in most of the previous animal studies. Aging and co-morbidities have been shown to modify or even abrogate the infarct size-limiting effects of interventions in animal experiments [6,11] and thus possibly underlie the negative results in clinical trials.

Of morbidities that potentially compromise the protective mechanisms of the heart, DM appears primarily important to study today. The number of patients with DM has been increasing worldwide in the past two decades, and these patients are predisposed to serious cardiovascular morbidity and mortality [12]. Despite recent progress in coronary intervention strategies, DM is associated with higher mortality after AMI due to more extensive atherosclerotic lesions and also hypertrophied and dysfunctional left ventricle [13-15]. Interestingly, Lamblin et al. recently showed that rates of cardiovascular death and heart failure after the first AMI were higher in DM patients than in non-DM patients, though ventricular function and remodeling were comparable in the two groups [16]. The increased mortality after AMI in DM patients is unlikely to be prevented simply by improvement of glycemic control, since intensive glycemic controls failed to reduce cardiovascular events as well as mortality rate in DM patients in recent large-scale clinical trials [17-19]. Hence, we need novel strategies for protecting DM patients with coronary artery diseases from excessive myocardial ischemia-reperfusion injury. To gain an insight into a novel strategy, we thoroughly review reported effects of DM on myocardial infarct size and on protective mechanisms against infarction and discuss future perspectives in this article. Our discussion on pathophysiology and clinical approach to diabetic cardiomyopathy was recently published elsewhere [20].

## Changes in myocardial susceptibility to infarction by DM

Clinical studies showed that DM increased the susceptibility of the myocardium to ischemia/reperfusion injury [21,22]. However, results of infarct size studies in diabetic animal models were contradictory (Tables 1 and 2), and the reason for the inconsistency remains unclear.

**Table 1:** Effect of diabetes mellitus on infarct size

**Species**	**DM**		**Index ischemia**		**First author**	**Journal**
	**type**	**duration**	**min**	**type**		
**Infarct size enlargement**						
Dog	2	75 d	120	regional, vivo	Forrat	Cardiovasc Res 1993
Rat (SD)	1	8 d	25	regional, vivo	Marfella	Diabetologia 2002
Rat (SD)	1	8 d	25	regional, vivo	Di Filippo	Diabetes 2005
Rat (SD)	1	30 d	30	global, vitro	Thirunavukkarasu	Free Radic Biol Med 2007
Rat (Wistar)	1	2 w	30	regional, vivo	Xiao	J Pharmacol Exp Ther 2004
Rat (Zucker obese)	2		30	regional, vivo	Jordan	J Pharmacol Exp Ther 2003
Rat (Zucker obese)	2	(10-12 w old)	30	regional, vivo	Katakam	Am J Physiol 2007
Rat (Zucker diabetic fatty)	2	(10-12 w old)	30	regional, vivo	Yue	Diabetes 2005
Rat (Zucker diabetic fatty)	2	(12-16 w old)	30	regional, vivo	La Bonte	Am J Physiol 2008
Rat (OLETF)	2	(25-30 w old)	20	regional, vivo	Miki	Diabetes 2009
Rat (OLETF)	2	(25-30 w old)	20	regional, vivo	Hotta	Circ Res 2010
Mice	1	8 d	25	regional, vivo	Marfella	Diabetes 2004
Mice	1	4 w	60	regional, vivo	Liu	Diabetes 2005
Mice (ob/ob)	2	(8-10 w old)	30	regional, vivo	Bouhidel	Am J Physiol 2008
Mice (ob/ob)	2	(8-10 w old)	30	regional, vivo	Calvert	Diabetes 2008
Mice (ob/ob)	2	(10-12 w old)	30	regional, vivo	Zhu	J Cell Mol Med 2011
Mice (KK-Ay)	2	(6 w old)	40	regional, vivo	Honda	J Mol Cell Cardiol 2008
**Infarct size reduction**						
Rabbit (NZ)	1	8 w	30	regional, vivo	Hadour	J Mol Cell Cardiol 1998
Rat (SD)	1	2 w	30	regional, vivo	Ma	N-S Arch Pharmacol 2006
Rat (Wistar)	2	11-12 m	30, 45	regional, vivo	Liu	Circulation 1993
Rat (Wistar)	1	1 w	30	regional, vivo	Ravingerová	Mol Cell Biochem 2003
Rat (Wistar)	1	4 w	30	regional, vivo	Xu	Exp Mol Pathol 2004
Rat (Wistar)	1	6 w	30	regional, vivo	Galagudza	Neurosci Behav Physiol 2007
Rat (Wistar)	2	10-12 w	30	global, vitro	Kravchuk	Exp Diabetes Res 2011
Rat (Wistar-Kyoto)	2	9 m	40	regional, vitro	Mozaffari	Hypertension 2003
Rat (Zucker diabetic fatty)	2	(16 w old)	50	regional, vitro	Kristiansen	Diabetologia 2004
Rat (Goto-Kakizaki)	2	(16 w old)	50	regional, vitro	Kristiansen	Diabetologia 2004
Rat (Goto-Kakizaki)	2	(16 w old)	45	regional, vitro	Kristiansen	Diabetologia 2011
**No change in infarct size**						
Dog	1	3 w	60	regional, vivo	Kersten	Am J Physiol 2000
Dog	1	3 w	60	regional, vivo	Kersten	Am J Physiol 2001
Rabbit (NZ)	1	10-16 w	30	regional, vivo	Vogel	Circ Res 1988
Rabbit (NZ)	1	4-5 w	60	regional, vivo	Nieszner	Exp Clin Endocrinol Diabetes 2002
Rabbit (NZ white)	1	5-6 w	30	regional, vivo	Ebel	Pflügers Arch 2003
Rat (SD)	1	6 w	30	regional, vivo	Ma	N-S Arch Pharmacol 2006
Rat (SD)	1	2 w	30	regional, vivo	Gross	Diabetes 2007
Rat (SD)	1	4 w, 8 w	30	global, vitro	Shi-Ting	Biomed Pharmacother 2010
Rat (SD)	1	4-5 w	30	regional, vivo	Drenger	Anesthesiology 2011
Rat (SD)	1	4 w	30	global, vitro	Okazaki	J Mol Cell Cardiol 2011
Rat (Wistar)	1	8 w	30	regional, vitro	Joyeux	Cardiovasc Res 1999
Rat (Wistar)	1	8 w	30	regional, vivo	Ravingerová	Mol Cell Biochem 2003
Rat (Wistar)	1	20 w	30	regional, vivo	Xu	Exp Mol Pathol 2004
Rat (Wistar)	1	4 w	25	global, vitro	Ghaboura	Basic Res Cardiol 2011
Rat (Goto-Kakizaki)	2		35	regional, vitro	Tsang	Diabetes 2005
Rat (Goto-Kakizaki)	2		35	regional, vitro	Bhamra	Basic Res Cardiol 2008
Rat (Goto-Kakizaki)	2	(12 w old)	35	regional, vivo	Bulhak	Am J Physiol 2009
Rat (Goto-Kakizaki)	2		30	regional, vivo	Matsumoto	Cardiovasc Drug Ther 2009
Mice	1	2 w	30	global, vitro	Przyklenk	Antioxid Redox Signal 2011
Mice (ob/ob)	2	(12-14 w old)	30	global, vitro	Przyklenk	Antioxid Redox Signal 2011

SD, Sprague–Dawley; NZ, New Zealand; OLETF, Otsuka Long-Evans-Tokushima fatty; d, days; w, weeks.

**Table 2:** Effect of hyperglycemia on infarct size

**Species**	**Duration of hyperglycemia**	**Blood sugar (mg/dl)**	**Index ischemia**		**First author**	**Journal**
			**min**	**type**		
**Hyperglycemia**						
**Infarct size enlargement**						
Dog	before I	585 vs. 71	60	regional, vivo	Kersten	Am J Physiol 1998
Rabbit (NZ white)	throughout	594 vs. 99	30	regional, vitro	Wong	J Diabetes Complications 2011
Rat (SD)	during I	324 vs 85	30	regional, vivo	Su	Am J Physiol 2007
**No change in infarct size**						
Dog	before I	310 vs. 71	60	regional, vivo	Kersten	Am J Physiol 1998
Dog	before I	310 vs. 75	60	regional, vivo	Kersten	Am J Physiol 2001
Dog	before I	316, 579 vs. 78	60	regional, vivo	Kehl	Anesthesiology 2002
Dog	before I	307 vs 83	60	regional, vivo	Gu	Anesthesiology 2008
Rabbit (NZ white)	before and during I	660 vs. 185	30	regional, vivo	Ebel	Pflugers Arch 2003
Rabbit (NZ white)	before I	257 vs 124	30	regional, vivo	Amour	Anesthesiology 2010
Rabbit (NZ white)	before and during I	338 vs 142	40	regional, vivo	Raphael	J Cardiovasc Pharmacol 2010
Rat (SD)	before and during I	360 vs 112	30	regional, vivo	Ichinomiya	Cardiovasc Diabetol 2012
Rat (Wistar)	before and during I	>400 vs. 121	25	regional, vivo	Weber	Eur J Pharmacol 2008
Rat (Wistar)	before and during I	459 vs 128	25	regional, vivo	Huhn	Br J Anaesth 2008
Rat (Wistar)	before and during I	355 vs 89	30	regional, vivo	Matsumoto	Cardiovasc Diabetol 2012
**Metabolic syndrome**						
**Infarct size enlargement**						
Rat (Wistar)	Western diet for 16 w	95 vs 87	40	regional, vitro	du Toit	Am J Physiol 2008
**No change in infarct size**						
Rat (SD)	high fat diet for 13 w	160 vs 144	45	regional, vivo	Thim	Clin Sci (Lond) 2006
Rat (WOKW)	(28 w old)	105 vs. ?	30	regional, vivo	Wagner	J Cardiovasc Pharmacol 2008

*NZ* New Zealand, *SD* Sprague–Dawley, *WOKW* Wistar-Ottawa-Karlsburg W, *before I* before ischemia, *during I* during ischemia, *w* weeks.

Multiple mechanisms are involved in cardiomyocyte necrosis induced by ischemia/reperfusion. Depletion of intracellular ATP, intracellular overloading with Na^+^ and Ca^2+^, and increased fragility of the cell membrane are major changes during ischemia relevant to cell necrosis. Accumulating evidence supports the notion that the mitochondrial permeability transition pore (mPTP) is primed during ischemia and is opened by Ca^2+^ overload and burst of reactive oxygen species at the time of reperfusion, leading to irreversible loss of mitochondrial functions and cell necrosis [23]. Theoretically, modification of ischemia-induced changes or reperfusion-induced opening of the mPTP should underlie the change in myocardial susceptibility to infarction in DM hearts. However, such modification has not been understood well.

### Experimental studies

Infarct size in animal models of DM was larger or smaller than or similar to, depending on reports, that in non-diabetic controls (Table 1). As shown in Table 1, there were numerous differences in experimental preparations and protocols, and a single factor cannot entirely explain the discrepancy in the effects of DM on infarct size. Duration of the diabetic state and plasma level of insulin (i.e., type 1 DM vs. type 2 DM) appear to influence myocardial tolerance against infarction. In a study by Ravingerová et al. [24], infarct size after 30-min ischemia was smaller in diabetic rat hearts at 1 week after streptozotocin (STZ) injection than in controls, but this infarct size limitation was not detected 8 weeks later. Two other studies have also shown that resistance of diabetic hearts to ischemia/reperfusion injury was detectable at the early phase of DM and disappeared later [25,26]. However, enlargement of infarct size was observed as early as 8 days after STZ injection [27-29], indicating that duration of DM is not the only factor responsible for inconsistent effects of DM on infarct size.

Difference in metabolic profiles associated with DM (i.e., presence or absence of hyperinsulinemia and types of dyslipidemia) may also be responsible for different changes in infarct size in DM animals. With a few exceptions [30,31], DM models with obesity and hyperinsulinemia showed increased myocardial susceptibility to ischemia/reperfusion-induced necrosis [32-41]. In contrast, high glucose level induced simply by glucose or dextrose infusion did not affect infarct size in the majority of studies, including two recent studies [42,43] (Table 2).

### Clinical studies

Increased myocardial susceptibility to infarction in DM patients was indicated by two clinical studies: infarct size determined by SPECT imaging after reperfusion therapy was larger by 30 ~ 70% in DM patients than in non-DM patients [21,22]. The change in myocardial tolerance to infarction is consistent with worse short- and long-term prognosis after AMI in DM patients [44,45]. Of particular relevance, Haffner et al. [46] showed that cardiovascular event risk in DM patients without prior myocardial infarction was comparable to the risk in non-diabetic patients with prior myocardial infarction. However, the untoward effects of DM on prognosis after AMI cannot be explained solely by increased myocardial vulnerability to ischemia/reperfusion injury, since the risk of heart failure and death was significantly higher in DM patients than in non-DM patients with comparable infarct sizes and left ventricular ejection fractions [47]. Myopathic changes (“diabetic cardiomyopathy”), impaired collateral recruitment, microcirculatory abnormalities, and underuse of evidence-based therapies are possible explanations, in addition to increased susceptibility to infarction, for detrimental prognosis after AMI in DM patients.

## Defects in intracellular protective signaling in DM hearts

DM is one of the co-morbidities that potentially modify myocardial response to protective interventions, including PC and PostC. A number of studies have been carried out to determine the efficacy of ischemic PC and PostC for protecting the myocardium in animal models of DM (Tables 3 and 4) and diabetic patients (Table 5). The majority of those studies showed that DM interferes with protective mechanisms of cardioprotective interventions. Myocardial protection by PC and PostC is achieved by activation of multiple protective signaling pathways that appear to converge to inhibit mPTP opening upon reperfusion via phosphorylation of glycogen synthase kinase-3β (GSK-3β) at Ser9 [6,8,9], and DM-induced defects in the protective signaling may be different depending on the model and/or phase of DM.

**Table 3:** Effect of diabetes mellitus on cardioprotection by pre- and post-conditioning

**Species**	**DM**		**PC stimuli**	**First author**	**Journal**
	**type**	**duration**			
**Preserved protection**					
**PC**					
Rat (SD)	1	4 w	3x 5I5R	Shi-Ting	Biomed Pharmacother 2010
Rat (Wistar)	2	11-12 m	3x 5I5R	Liu	Circulation 1993
Rat (Wistar)	1	6 w	LiCl 20 mM, IND 1 μM, SB 3 μM	Yadav	Mol Cell Biochem 2010
Rat (Zucker diabetic fatty)	2	(10-12 w old)	Rosiglitazone 3 mg/kg for 1 week	Yue	Diabetes 2005
Rat (OLETF)	2	(25-30 w old)	SB 1.2 mg/kg	Miki	Diabetes 2009
Rat (Goto-Kakizaki)	2		3x 5I10R	Tsang	Diabetes 2005
Rat (Goto-Kakizaki)	2	(12 w old)	WY 1 mg/kg	Bulhak	Am J Physiol 2009
Rat (Goto-Kakizaki)	2		olprinone 10 μg/kg	Matsumoto	Cardiovasc Drug Ther 2009
Mice (ob/ob)	2	(8-10 w old)	Metformin 125 μg/kg	Calvert	Diabetes 2008
Mice (KK-A^y^)	2	(6 w old)	Pioglitazone 25 mg/kg for 2 week	Honda	J Mol Cell Cardiol 2008
**PostC**					
Rat (SD)	1	2 w	SB 0.6 mg/kg	Gross	Diabetes 2007
Rat (Wistar)	1	4 w	SB 3 μM	Ghaboura	Basic Res Cardiol 2011
Rat (OLETF)	2	(25-30 w old)	BIO 0.08 mg/kg	Hotta	Circ Res 2010
Rat (Goto-Kakizaki)	2		Metformin 50 μM	Bhamra	Basic Res Cardiol 2008
Mice (ob/ob)	2	(8-10 w old)	Metformin 125 μg/kg	Calvert	Diabetes 2008
**Impaired protection**					
**PC**					
Dog	1	3 w	4x 5I5R	Kersten	Am J Physiol 2000
Dog	1	3 w	Diazoxide 2.5 mg/kg	Kersten	Am J Physiol 2001
Rabbit (NZ)	1	4-5 w	3x 2I2R	Nieszner	Exp Clin Endocrinol Diabetes 2002
Rat (SD)	1	8 w	3x 5I5R	Wang	Biomedicine Aging Pathol 2011
Rat (Wistar)	1	6 w	5I5R	Galagudza	Neurosci Behav Physiol 2007
Rat (Wistar)	1	6 w	4x 5I5R	Yadav	Mol Cell Biochem 2010
Rat (Wistar)	2	10-12 w	Metformin 200 mg/kg for 3 days	Kravchuk	Exp Diabetes Res 2011
Rat (Zucker obese)	2	(10-12 w old)	5I5R, Diazoxide 10 mg/kg	Katakam	Am J Physiol 2007
Rat (Zucker diabetic fatty)	2	(16 w old)	4x 2I3R	Kristiansen	Diabetologia 2004
Rat (OLETF)	2	(25-30 w old)	EPO 5000 U/kg	Miki	Diabetes 2009
Rat (OLETF)	2	(25-30 w old)	EPO 5000 U/kg, DADLE 1 mg/kg	Hotta	Circ Res 2010
Rat (Goto-Kakizaki)	2	(16 w old)	4x 2I3R	Kristiansen	Diabetologia 2004
Rat (Goto-Kakizaki)	2		5I10R, 2x 5I10R	Tsang	Diabetes 2005
Rat (Goto-Kakizaki)	2	(16-18 w old)	EPO 5000 U/kg	Miki	Diabetes 2009
Rat (Goto-Kakizaki)	2		isoflurane 1.0%	Matsumoto	Cardiovasc Drug Ther 2009
**PostC**					
Rat (SD)	1	2 w	Morphine 0.3 mg/kg	Gross	Diabetes 2007
Rat (SD)	1	4-5 w	3x 20sR20sI, sevoflurane 1.0 MAC	Drenger	Anesthesiology 2011
Rat (Wistar)	1	4 w	Darbopoetin alpha 5 μg/kg	Ghaboura	Basic Res Cardiol 2011
Mice	1	2 w	3x 10sR10sI, 6x 10sR10sI	Przyklenk	Antioxid Redox Signal 2011
Mice (ob/ob)	2	(8-10 w old)	6x 10sR10sI	Bouhidel	Am J Physiol 2008
Mice (ob/ob)	2	(12-14 w old)	3x 10sR10sI, 6x 10sR10sI	Przyklenk	Antioxid Redox Signal 2011
Mice (ob/ob)	2	(10-12 w old)	6x 10sR10sI	Zhu	J Cell Mol Med 2011
Mice (KK-A^y^)	2	(6 w old)	Pioglitazone 25 mg/kg	Honda	J Mol Cell Cardiol 2008
**Late PC**					
Rabbit (NZ white)	1	5-6 w	5I24hR	Ebel	Pflugers Arch 2003

*IND* indirubin-3 monooxime (a GSK-3β inhibitor), *SB* SB216763 (a GSK-3β inhibitor), *WY* 4-chloro-6-(2,3-xylidino)-2-pyrimidinylthioacetic acid (a PPAR-α agonist), *BIO* 6-bromo-indirubin-3'-oxime (a GSK3β inhibitor), *EPO* erythropoietin, *DADLE* [D-Ala2, D-Leu5]-enkephalin acetate (a δ-opioid receptor agonist), *5I5R* 5-min ischemia/5-min reperfusion, *5I24hR* 5-min ischemia/24-hour reperfusion, *10sR10sI* 10-second reperfusion/10-second ischemia, *MAC* minimal alveolar concentration, *w* weeks, *m* months.

**Table 4:** Effect of hyperglycemia on cardioprotection by pre- and post-conditioning

**Species**	**Duration of Blood sugar**		**PC stimuli**	**First author**	**Journal**
	**hyperglycemia**	**(mg/dl)**			
**Hyperglycemia**					
**Preserved protection**					
**PC**					
Dog	before I	397	Diazoxide 5.0 mg/kg	Kersten	Am J Physiol 2001
Dog	before I	291	Isoflurane 1.0 MAC	Kehl	Anesthesiology 2002
Rat (SD)	before and during I	360	Fasudil 0.5 mg/kg	Ichinomiya	Cardiovasc Diabetol 2012
Rat (Wistar)	before and during I	446	Sevoflurane 1.0 MAC + CysA 5 mg/kg	Huhn	Br J Anaesth 2008
Rat (Wistar)	before and during I	343, 447	MIL 30 μg/kg, LEVO 100 μg/kg	Matsumoto	Cardiovasc Diabetol 2012
**Impaired protection**					
**PC**					
Dog	before I	296	4x 5I5R	Kersten	Am J Physiol 1998
Dog	before I	329	Diazoxide 2.5 mg/kg	Kersten	Am J Physiol 2001
Dog	before I	581, 558	Isoflurane 0.5, 1.0 MAC	Kehl	Anesthesiology 2002
Dog	before I	293	4x 5I5R	Gu	Anesthesiology 2008
Rabbit (NZ white)	before I	281	Isoflurane 1.0 MAC	Amour	Anesthesiology 2010
Rat (Wistar)	before I or during I	>400	Desflurane 1.0 MAC	Weber	Eur J Pharmacol 2008
**PostC**					
Rabbit (NZ white)	before and during I	353	Isoflurane 1.0 MAC	Raphael	J Cardiovasc Pharmacol 2010
Rat (SD)	before and during I	360	Diazoxide 10 mg/kg, Fasudil 0.15 mg/kg	Ichinomiya	Cardiovasc Diabetol 2012
Rat (Wistar)	before and during I	445, 441	Sevoflurane 1.0 MAC, CysA 5 mg/kg	Huhn	Br J Anaesth 2008
**Metabolic syndrome**					
**Impaired protection**					
**PostC**					
Rat (WOKW)	(28 w old)	105	3x 30sI30sR	Wagner	J Cardiovasc Pharmacol 2008

*NZ* New Zealand, *SD* Sprague–Dawley, *WOKW* Wistar-Ottawa-Karlsburg W, *before I* before ischemia, *during I* during ischemia, *MAC* minimal alveolar concentration, *CysA* cyclosporine A, *MIL* milrinone, *LEVO* levosimendan, *5I5R* 5-min ischemia/5-min reperfusion, *30sR30sI* 30-second reperfusion/30-second ischemia.

**Table 5:** Effect of ischemic postconditioning in patients with acute myocardial infarction

**First author**	**% of DM**	**Age**	**Protocol**	**Results**			
				**ST resolution**	**LV function**	**Enzyme release**	**Infarct size**
**Ischemic PostC**							
Staat	20 vs. 13	58 vs. 56	4x 60sR/I	Improved	N/A	Reduced (peak, AUC)	N/A
Laskey	N/A	58 vs. 58	2x 90sR/I	Improved	N/A	Nochange (peak)	N/A
Ma	38 vs. 45	64 vs. 64	3x 30sR/I	N/A	Improved WMSI	Reduced (peak)	N/A
Yang	26 vs. 28	59 vs. 63	3x 30sR/I	No change	No change	Reduced (AUC)	Reduced (SPECT, 1w)
Thibault	12 vs. 10	56 vs. 56	4x 60sR/I	N/A	Improved EF, WMSI	Reduced (AUC)	Reduced (SPECT, 6m)
Laskey	38 vs. 42	60 vs. 58	2x 90sR/I	Improved	No change	Reduced (peak)	N/A
Xue	21 vs. 29	54 vs. 62	4x 60sR/I	Improved	Improved EF, WMSI	Reduced (peak, AUC)	Reduced (SPECT, 1w)
Lønborg	7 vs. 7	61 vs. 62	4x 30sR/I	No change	No change	N/A	Reduced (CMR, 3m)
Sörensson	25 vs. 17	63 vs. 62	4x 60sR/I	N/A	No change *1	No change (AUC)	No change (CMR, 1w) *1
Freixa	23 vs. 17	59 vs. 60	4x 60sR/I	N/A	No change	No change (peak)	No change (CMR, 1w & 6m)
Tarantini	18 vs. 3	60 vs. 60	4x 60sR/I	No change	No change	No change (AUC)	No change (CMR, 1m)

*DM* diabetes mellitus, *MBG* myocardial blush grade, *CF* coronary flow, *WMSI* wall motion score index, *EF* ejection fraction, *AUC* area under the curve, *MACE* major adverse cardiac events, *SPECT* single photon emission computed tomography, *CMR* cardiovascular magnetic resonance, *w* week, *m* month;

*1 Improved by postconditioning with large area at risk.

### Experimental studies

With a few exceptions, previous studies demonstrated that cardioprotection achieved by ischemic PC or PostC is impaired in DM: the infarct size-limiting effect was lost or required extra cycles of ischemia/reperfusion in experimental diabetes (Table 3). Tsang et al. [48] reported that one, two and three cycles of ischemic PC significantly reduced infarct size in normal rats; however, three cycles of ischemic PC were required to limit infarct size in diabetic Goto-Kakizaki rats. Their findings suggested that the threshold for PC protection is increased in the diabetic myocardium. Liu et al. [49] were the first to examine the effects of DM on PC and they reported preservation of PC protection in STZ-induced DM. However, since they used a PC protocol with multiple cycles of ischemia/reperfusion only, the change in the threshold for inducing PC protection might have been missed in their study. In fact, impairment of PC by DM has been observed in multiple species (rats, rabbits and dogs) and different models of DM (STZ-induced type 1 DM and genetic models of type 2 DM) [30,37,50-54]. Mimetics of ischemic PC and PostC (diazoxide, erythropoietin, [D-Ala^2^, D-Leu^5^-enkephalin acetate [DADLE] and isoflurane) were also ineffective for limitation of infarct size in DM hearts [33,34,37,55-59], confirming that DM impairs intracellular signaling mechanisms relevant to myocardial protection.

Several steps in cytoprotective signaling have been found to be disrupted by DM in the heart. Blocked or attenuated signaling via phosphoinsitide-3 kinase (PI3K)-Akt, extracellular signal-regulated kinase (ERK) and/or signal transducer and activator of transcription-3 (STAT3) pathways was detected in association with loss or reduced efficiency of PC in protection against infarction [31,33,34,36,48,57-59]. Our recent studies have shown that phosphorylation of Jak2, being upstream of PI3K-Akt signaling, is inhibited by enhanced calcineurin activity and that phosphorylation of GSK-3β by ERK is lost by an endoplasmic reticulum stress-dependent mechanism in OLETF, a rat model of obese type 2 DM [33,34]. Furthermore, protein level of active GSK-3β, a pro-necrotic and pro-apoptotic kinase, was increased in mitochondria, leading to reduction in the threshold for mPTP opening in response to Ca^2+^ overload [34]. On the other hand, a protective mechanism downstream of GSK-3β phosphorylation appears to be intact in diabetic hearts, since direct pharmacological inhibitors of GSK-3β limited infarct size in diabetic hearts as in non-diabetic animals [33,34,54,57,58].

An important and clinically relevant issue is whether hyperglycemia per se is responsible for disruption of protective signaling in PC and PostC. Acute hyperglycemia induced by dextrose infusion attenuated infarct size limitation by ischemic PC, a mitochondrial ATP-sensitive K^+^ channel (K_ATP_ channel) opener and anesthetic agents (Table 4). These results indicate a primary role of hyperglycemia in impairment of protective signaling. Interestingly, Przyklenk et al. [31] reported that the cardioprotective effect of ischemic PostC was re-established in STZ-induced diabetic mice by pancreas islet cell transplantation. Transplantation of islet cells in diabetic mice normalized both blood glucose level and free fatty acid level and cellular signaling, such as ERK phosphorylation, activated by ischemic PostC. Since dyslipidemia was also shown to attenuate the infarct size-limiting effect of ischemic PC [60,61], restoration of myocardial response to PostC in the diabetic heart by islet cell transplantation might be achieved by normalization of both plasma glucose level and lipid profile. Nevertheless, circumstantial evidence in animal experiments supports the notion that normalization of the metabolic profile restores protective signaling mechanisms in the DM heart.

### Clinical studies on PC

PC protection in human hearts has been demonstrated by results of in vitro experiments using human ventricular myocytes [62] and atrial trabeculae [63] and by detailed analyses of myocardial responses in patients with naturally occurring ischemic syndromes [64,65]. Since ischemic PC and PC-like phenomena in human hearts have been recently reviewed elsewhere [7,65], we briefly summarize the results that are relevant to the effects of DM on ischemic PC. As for PC-like effects of preinfarct angina, different groups reported that pre-infarct angina was associated with smaller infarct size determined by creatine kinase (CK) release, improved left ventricular function and reduced mortality [64,66]. Furthermore, in overall analysis of AMI patients, angina before AMI was associated with better long-term prognosis [67,68]. However, in patients with DM, beneficial effects of pre-infarct angina were not detected [69]. Ishihara et al. [69] showed that CK release and recovery of cardiac function and in-hospital mortality after AMI were similar in DM patients with and without pre-infarct angina. DM-induced impairment of ischemic PC protection in human hearts has also been indicated by studies in which myocardial injury was assessed during angioplasty [70] and during a treadmill exercise test [71] by use of electrocardiography.

Direct evidence for diabetes-induced loss of ischemic PC protection in the human myocardium was provided by an in vitro experiment using atrial trabeculae obtained at open heart surgery. CK release and contractile dysfunction after hypoxia/reoxygenation in vitro was significantly suppressed by PC in trabeculae from non-diabetic patients but not in trabeculae from diabetic patients [63,72].

### Clinical studies on PostC

In contrast to PC, PostC or administration of its mimetic at the time of reperfusion is clinically feasible. In 2005, Staat et al. [73] translated for the first time the concept of ischemic PostC into the clinical setting. PostC with four cycles of 1-min inflation and 1-min deflation before full reperfusion successfully reduced CK release in a small and selected cohort of patients with AMI. Efficacy of ischemic PostC in patients with AMI was subsequently confirmed in a number of small-scale studies, using as endpoints cardiac biomarkers, ECG ST-segment resolution, and anatomic infarct size (Table 5). However, three recent studies failed to show protection by ischemic PostC in terms of CK and troponin release, LV function or infarct size that was determined by delayed enhancement magnetic resonance imaging (MRI) [74-76]. The reasons for the discrepant results remain uncertain, but there are some possibilities: differences in the protocols of ischemic PostC, presence of co-morbidities and use of different pharmacological agents for the co-morbidities.

In the PostC protocol, duration from reperfusion to the first re-occlusion and number of re-occlusions have been shown to influence the infarct size-limiting effects of PostC in animal models of AMI [77-79]. Retrospective analyses by Darling et al. [80] and Wang et al. [81] suggest that larger numbers of balloon inflations–deflations were associated with greater protection in patients with AMI. However, an appropriate algorithm of PostC stimulus to maximally protect the human myocardium has not been established. In addition, protection by PostC in animal models of AMI is attenuated with aging and presence of co-morbidities, such as DM, hypertension and hypercholesterolemia [6,11]. Although characteristics of patients, such as age and prevalence of DM, seem to be similar in the human studies (Table 5), difference in durations of the co-morbidities might have been involved in the different outcomes. Nevertheless, the effect of DM on protection afforded by PostC has not been specifically examined in clinical studies.

The effects of pharmacological PostC (i.e., administration of pharmacological agents at reperfusion) on myocardial injury have been examined in a number of clinical trials. Results of the trials are a mixture of positive and negative results, and the reason for the discrepancy remains unknown [82-92]. Adenosine infusion limited infarct size, but neither the death rate during 6-month follow-up nor the rate of re-hospitalization for heart failure were reduced by the adenosine treatment [82,84]. A *post hoc* analysis suggested that only patients with early reperfusion therapy received clinical benefits of adenosine, such as a reduction in death or heart failure [85]. In the J-WIND trial [86], the effects of nicorandil and ANP on infarct size in different study arms were examined. Administration of nicorandil failed to significantly limit infarct size, though recombinant ANP infusion was shown to induce a small but significant reduction in infarct size. The results of studies on the effect of erythropoietin administered at reperfusion were discrepant [87-92]. Taken together, there are a few promising agent for infarct size limitation in AMI patients, but none of their effects has been unequivocally demonstrated to be sufficient for improving prognosis.

## Effects of anti-diabetic medications on AMI in diabetic patients

Possible adverse effects of sulfonylureas on cardiac events in DM patients have been debated for decades, and recent studies have indicated that cardiovascular risk was significantly higher in DM patients treated with sulfonylureas than in those who received metformin monotherapy [93,94]. Sulphonylureas have been shown to inhibit protection of PC and PostC by blocking the ATP-sensitive K^+^ channel (K_ATP_ channel) in cardiomyocytes, raising concern that such actions of sulphonylureas may be detrimental at the time of AMI in DM patients. However, the effects on PC and PostC are actually not uniform across sulfonylureas. Glibenclamide, a non-specific blocker of the K_ATP_ channel, abrogated the infarct size-limiting effect of PC and PostC in non-diabetic animals [6,7,95]. In contrast, glimepiride, a second-generation sulfonylurea, did not block the protective effect of PC in the animal myocardium or human myocardium [96,97], presumably because this agent has few cardiac actions [98]. In addition, glimepiride was shown to have a PostC-mimetic action by activation of PI3K/Akt in the rabbit myocardium [99]. However, the effects of sulfonylureas on PC in diabetes have been tested in only a few studies [51,96], since PC and PostC failed to limit infarction in most of the DM models. Interestingly, Nieszner et al. [51] reported that infarct-sparing effect of PC in alloxan-treated diabetic rabbits was restored by pretreatment with a low dose, but not a high dose, of glibenclamide. The mechanism by which glibenclamide restored myocardial response to PC in the DM rabbit is unclear. However, beneficial effects of glycemic control, possibly on PI3K/Akt signaling, might have overwhelmed the effect of partial inhibition of cardiac K_ATP_ channels when an appropriate dose of glibenclamide was selected.

Metformin is an insulin-sensitizing agent that reduces hepatic glucose output and increases the uptake of glucose in peripheral tissues, including the skeletal muscle. In non-diabetic animals, metformin reduced infarct size by activation of AMP-activated protein kinase and/or Akt [95]. However, whether metformin is additive to ischemic PC or PostC has not been examined. Infarct size-limiting effect of metformin in diabetic animals was controversial: there are two positive reports [40,100] and one negative report [101].

Thiazolidinediones (pioglitazone, rosiglitazone) administered before ischemia reduced infarct size in both normal and diabetic animals [39,41,95]. However, the impact of the cardioprotective effect of thiazolidinediones on clinical outcome is unclear, since the effects of pioglitazone and rosiglitazone on risk of cardiovascular events appear discrepant [102].

In the past few years, growing evidence has demonstrated cardiovascular effects of incretin-based therapy using glucagon-like peptide-1 (GLP-1) receptor agonists and inhibitors of dipeptidyl peptidase-4 (DPP-4) [95,103-105]. However, the effects of GLP-1 receptor agonists or DPP-4 inhibitors on ischemia/reperfusion injury have not been examined in diabetic animals except for a study by Huisamen et al. [106] They reported that enlargement of infarct size in obese pre-diabetic rats was reduced by treatment with a DPP-4 inhibitor for 4 weeks [106]. Further investigation is necessary for clarifying the effects of GLP-1 analogues and DPP-4 inhibitors on intracellular protective signaling and its modification by DM, though there are a number of on-going large-scale clinical trials to test the effects of incretin-based therapy on cardiovascular outcomes [107].

## Conclusions and perspectives

DM potentially increases myocardial susceptibility to ischemia/reperfusion injury, though it cannot be explained by chronic hyperglycemia alone. DM also modifies myocardial responses to ischemic and pharmacological PC and PostC by disruption of intracellular signaling responsible for enhancement of resistance to cell death. These alterations in the diabetic heart appear to underlie the poor prognosis of DM patients after AMI. In addition, a defect of the protective mechanisms caused by aging, DM and possibly other co-morbidities is one of possible reasons why infarct size-limiting effects of agents in animal studies could not have been translated into AMI patients.

Analyses of modifications of cytoprotective signaling by DM, heart failure, dyslipidemia and hypertension generally showed that defects in the signaling by the pathological factors are upstream of a step of GSK-3β phosphorylation and mPTP regulation [6,9]. Hence, a reasonable approach to protect the heart in DM patients is manipulation of protective mechanisms at the level of GSK-3β or mPTP. Blockade of mPTP opening by cyclosporine A at the time of reperfusion is one such approach, and its effect was first examined in 58 AMI patients [108]. Infarct size determined by MRI at 5 days after AMI was significantly limited by cyclosporine A and the beneficial effects persisted until 6 month after AMI [109]. The results are very promising and support the feasibility of a strategy for cardioprotection targeting the mPTP. However, how DM modifies the mPTP regulation and how we can suppress opening of the mPTP at the time of reperfusion need to be further investigated for devising a novel therapy for cardioprotection in DM patients.

## Abbreviations

AMI: Acute myocardial infarction; CK: Creatine kinase; DADLE: [D-Ala2,D-Leu5]-enkephalin acetate; DM: Diabetes mellitus; DPP-4: Dipeptidyl peptidase-4; ERK: Extracellular signal-regulated kinase; GLP-1: Glucagon-like peptide-1; GSK-3β: Glycogen synthase kinase-3β; IHD: Ischemic heart disease; KATP channel: ATP-sensitive K+ channel; MPTP: Mitochondrial permeability transition pore; MRI: Magnetic resonance imaging; PC: Preconditioning; PCI: Percutaneous coronary interventions; PostC: Postconditioning; PI3K: Phosphoinositide-3 kinase; PKC: Protein kinase C; STZ: Streptozotocin.

## Competing interests

TaM has received research funding from Daiichi-Sankyo, Japan, Eisai, Japan, and Pfizer, Japan. TI and DS have no conflicts of interest or financial ties to disclose. TeM has received research funding from Takeda Pharmaceuticals, Japan, Novartis, Japan, Chugai Pharmaceutical, Japan, and Astellas Pharma, Japan.

## Authors' contributions

TaM collected and arranged the data in sequence, drafted and correspond the manuscript. TI and DS contributed to collection and analysis of the data. TeM contributed to analysis and interpretation of data and revision. All authors have read and approved the final manuscript.
